# Mild hypoxia-induced structural and functional changes of the hippocampal network

**DOI:** 10.3389/fncel.2023.1277375

**Published:** 2023-09-29

**Authors:** Alexandra Hencz, Andor Magony, Chloe Thomas, Krisztina Kovacs, Gabor Szilagyi, Jozsef Pal, Attila Sik

**Affiliations:** ^1^Institute of Physiology, Medical School, University of Pecs, Pecs, Hungary; ^2^Institute of Transdisciplinary Discoveries, Medical School, University of Pecs, Pecs, Hungary; ^3^Institute of Clinical Sciences, College of Medical and Dental Sciences, University of Birmingham, Birmingham, United Kingdom; ^4^Institute of Biochemistry and Medical Chemistry, Medical School, University of Pecs, Pecs, Hungary

**Keywords:** hippocampus, mild hypoxia, dark neuron, electrophysiology, network oscillation

## Abstract

Hypoxia causes structural and functional changes in several brain regions, including the oxygen-concentration-sensitive hippocampus. We investigated the consequences of mild short-term hypoxia on rat hippocampus *in vivo*. The hypoxic group was treated with 16% O_2_ for 1 h, and the control group with 21% O_2_. Using a combination of Gallyas silver impregnation histochemistry revealing damaged neurons and interneuron-specific immunohistochemistry, we found that somatostatin-expressing inhibitory neurons in the hilus were injured. We used 32-channel silicon probe arrays to record network oscillations and unit activity from the hippocampal layers under anaesthesia. There were no changes in the frequency power of slow, theta, beta, or gamma bands, but we found a significant increase in the frequency of slow oscillation (2.1–2.2 Hz) at 16% O_2_ compared to 21% O_2_. In the hilus region, the firing frequency of unidentified interneurons decreased. In the CA3 region, the firing frequency of some unidentified interneurons decreased while the activity of other interneurons increased. The activity of pyramidal cells increased both in the CA1 and CA3 regions. In addition, the regularity of CA1, CA3 pyramidal cells’ and CA3 type II and hilar interneuron activity has significantly changed in hypoxic conditions. In summary, a low O_2_ environment caused profound changes in the state of hippocampal excitatory and inhibitory neurons and network activity, indicating potential changes in information processing caused by mild short-term hypoxia.

## Introduction

The presence of oxygen (O_2_) is essential for the brain to maintain basic physiological functions, as neurons selectively produce their energy through aerobic production (Rolfe and Brown, 1997; Özugur et al., 2020). Consequently, O_2_ levels which deviate from physiological concentrations (21% O_2_) can significantly affect the metabolic efficiency of neurons (Malthankar-Phatak et al., 2008; Vestergaard et al., 2016). Reduced bioavailability of O_2_ in the brain and various tissues can result in hypoxic conditions (Connett et al., 1990). Hypoxia plays a fundamental role in normal physiological conditions, such as in vertebrate embryonic development and stem cell regulation. Activity-induced hypoxia can regulate adaptive gene expression and drive neuroplasticity (Dunwoodie, 2009; Mohyeldin et al., 2010; Wakhloo et al., 2020; Butt et al., 2021). However, hypoxia may also be undesirable and lead to pathological conditions (Marti et al., 2000; Kent et al., 2011; Ahmad et al., 2012; Smedley and Grocott, 2013; Tsui et al., 2014; Yan et al., 2014; Ferdinand and Roffe, 2016). It can be caused by many diseases, such as severe anaemia, obstructive sleep apnea syndrome, chronic obstructive pulmonary disease or COVID-19 (Marti et al., 2000; Kent et al., 2011; Ahmad et al., 2012; Tsui et al., 2014; Rahman et al., 2021). Hypoxia can develop after stroke or traumatic brain injury, resulting in prolonged neuritis, increased extravasation of biomarkers and poor clinical and functional outcomes (Yan et al., 2014; Ferdinand and Roffe, 2016). People exploring high altitudes for recreation or work are exposed to hypoxia (hypobaric hypoxia), which can cause high-altitude illness (Smedley and Grocott, 2013). Several studies have shown that in hypobaric hypoxia, even acute, mild hypoxia can have a negative effect on cognitive functions. Minimal impairments of neuropsychological functioning may already be detected at 16.4% O_2_ (~2000 m) (Virués-Ortega et al., 2004). Smith (2013) reported that the light sensitivity of the dark-adapted eye decrements at 17.2% O_2_ (1,524 m altitude), short- and long-term memory impairment appears at 15.4% O_2_ (2,438 m altitude) and the performance on previously learned encoding and conceptual reasoning tasks decreases at 14.2% O_2_ (3,048 m eltitude) (Smith, 2013). Based on previous research, hypobaric hypoxia can cause more significant damage compared to normobaric hypoxia because it leads to greater hypoxemia, hypocapnia, blood alkalosis and lower O_2_ arterial saturation (Savourey et al., 2003). However, the effect of normobaric, mild hypoxia cannot be neglected either, because the body tries to adapt to the lower O_2_ level, which can cause serious damage (Chen et al., 2020).

Similarly, even mild hypoxia-ischemia can produce disproportionately harmful effects, as observed in preterm fetuses (Galinsky et al., 2018). Neonatal hypoxic ischemia is the major cause of mortality and disability in human neonates (Grow and Barks, 2002; Ferriero, 2004; Shalak and Perlman, 2004) and is responsible for 23% of infant mortality (Grow and Barks, 2002; Ferriero, 2004; Shalak and Perlman, 2004; Lawn et al., 2005). It also causes early and delayed neurodegeneration in the developing brain (Northington et al., 2001). Highly metabolically active areas of the brain, such as the neocortex, striatum, and hippocampus (CA1 region), are susceptible to insufficient blood flow (Rice et al., 1981; Koroleva and Vinogradova, 2000). It is well known, that a reduced amount of blood flow (hypoperfusion) induces oxidative stress leading to cell death, especially in the vascular endothelium and in a selective population of neurons with high metabolic activity (Aliev et al., 2014). It has been demonstrated that changes in brain oxygen metabolism and impaired mitochondrial function are the key players in several neurodegenerative diseases progression, such as Alzheimer’ disease, Parkinson’s disease, Huntington’s disease or progressive supranuclear palsy (Browne et al., 1999; Park et al., 2001; Aliev et al., 2004; Hwang, 2013; Aliev et al., 2014). Insufficient O_2_ and glucose supply to the highly metabolically active hippocampal neurons can cause damage in a short time frame (Watts et al., 2018; Grube et al., 2021). This condition causes structural destabilization of the hippocampal neural circuits, which can lead to impairment of hippocampal-mediated learning and memory mechanisms (De Jong et al., 1999; Liu et al., 2005; Farkas et al., 2006; Maiti et al., 2007; Melani et al., 2010; Lana et al., 2013). In the hippocampus, pyramidal neurons in the CA1 region are most sensitive to damage caused by hypoxia-ischemia, while neurons in the CA3 region and dentate gyrus are more resistant (Kawasaki et al., 1990; Schmidt-Kastner et al., 1990; Hsu et al., 1994; Kreisman et al., 2000).

The oxygen consumption is highest in hippocampal subfield CA3 and the oxygen consumption is high during gamma oscillations (~30–80 Hz) (Kann et al., 2011). Thus, although a subtle decrease in the interstitial partial pressure of O_2_ does not significantly affect the viability of CA3 neuron populations, it can disrupt the interaction between the activity of excitatory pyramidal cells and fast-spiking interneurons and cause a decrease in the resulting gamma oscillations (Huchzermeyer et al., 2008; Kann et al., 2011). The effects of chronic intermittent hypoxia may be specifically detrimental to central nervous system (CNS) function, specifically due to the overactivation of N-methyl-D-aspartate (NMDA) receptors, which can lead to overload dephosphorylation of intracellular calcium and extracellular signal-regulated kinases (Wang et al., 2015). Experimental studies revealed reduced synaptic transmission and excitability in CA1 neurons due to ischemic hypoxia *in vivo* (Chi and Xu, 2000). Furthermore, in combination with inflammation, hypoxia has been shown to reduce synaptic signaling and excitability in CA1 neurons in the hippocampus, whereas reoxygenation can cause excessive excitability in these CA1 neurons (Yang et al., 2018).

Collectively, studies suggest the vulnerability of the hippocampus due to hypoxic ischemia. Ischemia describes a lack of blood supply, meaning that glucose and essential nutrient levels are also reduced in addition to O_2_. In this present study, our primary goal is to elucidate the underlying mechanisms of the brain’s vulnerability to normobaric hypoxia, including the effect of acute hypoxia on hippocampal network activity and to investigate the hippocampal interneuronal subtypes to determine which are vulnerable or resistant to hypoxic conditions.

## Materials and methods

### Animals and experimental procedure of hypoxic exposure

The experiments were performed on 40 male Wistar rats (Charles River, Hungary) weighing 250-280 g at the time of surgery. The animals were kept in a temperature (21 ± 2°C) and light-controlled room (12:12-h light–dark cycle, with lights on at 7:00). Standard laboratory food pellets (CRLT/N Charles River Kft, Budapest, Hungary) and tap water were available *ad libitum*.

All animal experiments were conducted following guidelines and protocols approved by the National Ethical Council for Animal Research (Permit number: BA/73/0052–5/2022, Hungary). They were by the directive of the European Communities Council on the protection of animals used for scientific purposes (Directive 2010/63/EU of the European Parliament and the Council).

Rats were randomly divided into the following experimental groups: (a) Animals were exposed to different O_2_ levels for 1 h, either normoxic (21% O_2_) or hypoxic (16% O2) conditions at normal ambient pressure. Oxygen levels in the induction chamber were continuously monitored with an O_2_ sensor (R17 MED, Viamed Limited, United Kingdom). After O_2_ treatment, the rats were anaesthetized for histological examination by intraperitoneal injection of urethane (1.5–2.0 g/kg, Sigma, St. Louis, MO, United States).

(b) For electrophysiological testing, the animals were examined under anesthesia (urethane intraperitoneal injection 1.1–1.3 g/kg; Sigma, St. Louis, MO, United States) using 32-channel probes (A4x8-5 mm-100-200-177, NeuroNexus Technologies, Inc., United States). In the first step, we recorded the baseline under 21% O_2_ exposure and then reduced it to 16% O_2_ level. The animals were kept in this O_2_ environment for 1 h, and during the last 15 min, a continuous electrophysiological recording was performed.

### Histology

#### Silver impregnation method (Gallyas staining)

After 1 h at normoxic (21%) or hypoxic (16%) O_2_ exposure, the rats (21% *n* = 10, 16% *n* = 10) were anaesthetized by intraperitoneal injection of urethane (1.5–2.0 g/kg). Transcardial perfusion with 4% paraformaldehyde (PFA) in 0.1 M phosphate-buffered saline (PBS) was performed immediately after euthanasia, and later the brains were post-fixed in 4% PFA in PBS. The brains were cut into coronal slices (50 μm) using a vibratome (Vibratome® Series 3,000; Technical Products International Inc., St Louis, MO), and the slices were stored in Tris-buffer (pH = 7.4) at 4°C. Histological examinations were performed in three consecutive (Anterior–Posterior -4 mm) sections according to the atlas of Paxinos and Watson (Paxinos and Watson, 2006).

A special silver method (Gallyas method) was used to detect the compaction of ‘dark’ neurons (Gallyas et al., 1990), which are labeled at a very early decision stage of degeneration (Gallyas et al., 1993). Briefly, brain slices were subjected to a series of dehydration steps and incubated for 16 h at 56°C in 1% sulfuric acid in 1-propanol (esterification). Sections were rehydrated in a series between 100 and 50% 1-propanol (1–2 min each), followed by washing with double-distilled water for 5 min and treated with 1% acetic acid for 5 min. The slices were immersed in the silver staining solution, and then 1% acetic acid was added to stop the reaction.

#### Immunohistochemistry

Brain slices were immunostained with antibodies against interneuron markers. Briefly, background antigenicity was blocked with 2% normal serum (Goat, Vector Laboratories, Inc., Burlingame, CA), and cell membranes were permeabilized with 1% Triton X-100 (Sigma Aldrich, Dorset, United Kingdom) in 0.1 M Phosphate Saline, pH 7.4 (PBS) for 2 h at room temperature (22-25°C). The blocking solution was removed, and primary antibody solution (primary antibody, 2% normal horse serum, 1% Triton X-100 in PBS) was added for Somatostatin (1: 100, USCN Life Science Inc., Hubei, China), Parvalbumin (1:500, Sigma-Aldrich, Dorset, United Kingdom), Neuropeptide Y (1:4000, Immuno STAR Inc., Hudson, WI) Cholecystokinin (1:100, USCN Life Science Inc., Hubei, China), Calretinin (1:100, USCN Life Science Inc., Hubei, China), Calbindin (1:100, USCN Life Science Inc., Hubei, China), Caspase-3 (1:50, USCN Life Science Inc., Hubei, China) was added and incubated overnight at 4°C. The next day, the slices were washed three times in PBS. Secondary antibodies (1:500, Alexa Fluor ® 488 anti-rabbit IgG Molecular Probes Life Technologies, Paisley, United Kingdom; Alexa Fluor ® 546 anti-mouse IgG Molecular Probes Life Technologies, Paisley, United Kingdom, Alexa Fluor ® 488 anti-mouse IgG Molecular Probes Life Technologies, Paisley, United Kingdom) were diluted in PBS, and the slices were incubated at room temperature for 2 h. The slices were washed three times in PBS and mounted with Mowiol® medium. Slides were viewed with an epifluorescence microscope (Olympus BX61 TRF, Tokyo, Japan) and excited at 546 nm or 488 nm, depending on the fluorochrome used.

#### Surgery and electrophysiological recording

Rats (*n* = 10) were placed in a stereotaxic frame on top of an electric heating pad under urethane anaesthesia, and an O_2_ gas mixture was administered through an anaesthetic mask. Rectal temperature was continuously monitored, body temperature was maintained within 37.0 ± 0.5°C, and respiratory and heart rates were also continuously monitored to ensure adequate levels of anaesthesia.

**Figure 1:**
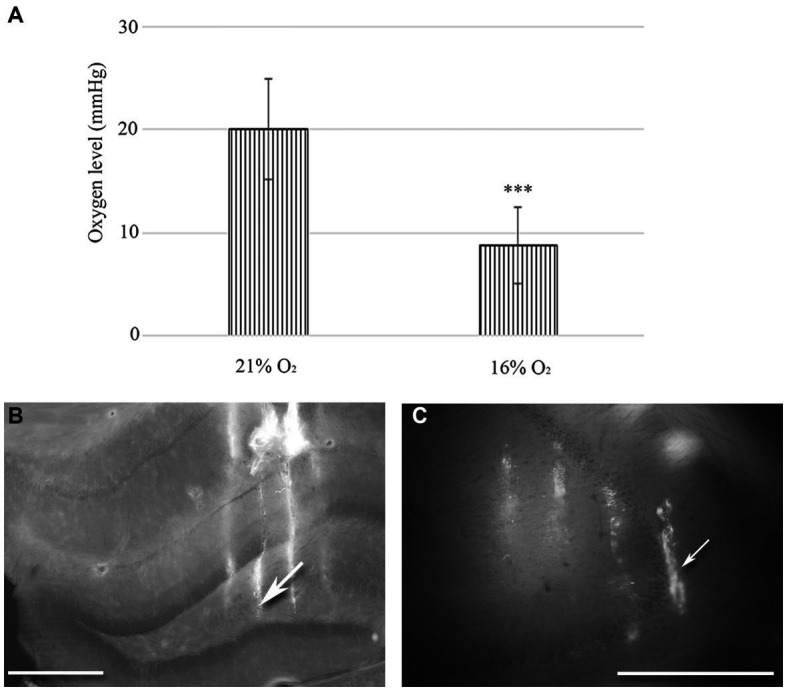
**(A)** Changes in tissue O_2_ levels were measured in the hippocampal regions of urethane-anaesthetized animals (*n* = 20). There is a statistically significant difference between normoxic (21%) and hypoxic (16%) conditions. Error bars represent mea*n* ± SEM. ***p < 0.001 The location of 32-channel probes in CA1-hilus axis **(B)** and in the CA3b region **(C)**. Traces of DiI-coated 4 shanks are visible in the fluorescent image. Scale bar: 600 μm.

To measure the activity of hippocampal neurons, 32-channel probes (A4x8-5 mm-100-200-177, NeuroNexus Technologies, Inc., United States) were implanted under sterile conditions. An incision was made between the eyes to the back of the skull. After cleaning the skull, a circular hole was made with a 2 mm diameter drill (Hilus-CA1 region: Medial-Lateral 1.2–2.2 mm, Anterior–Posterior -4 mm, CA3 region: Medial-Lateral 3.6–4.6 mm, Anterior–Posterior -4 mm) (Paxinos and Watson, 2006). Dura mater was removed, and a 32-channel electrode array was dipped in a 2% DiI solution (Sigma-Aldrich) before being inserted into the hippocampus. The probes were inserted into the brain, avoiding the blood vessels (Figures 1B,C). The probes were attached to a micromanipulator to allow precise vertical movement to the desired depth position (Hilus-CA1 region: Medial-Lateral 1.4–2.0 mm, Anterior–Posterior -4 mm, Dorsal Ventral −3.6 mm, CA3 region: Medial-Lateral 3.8–4.4 mm, Anterior–Posterior -4 mm, Dorsal-Ventral -4 mm) according to the atlas of Paxinos and Watson (Paxinos and Watson, 2006). During the experiment, the brain’s surface was covered with a saline solution. Brain tissue oxygenation was monitored with a 10 μm diameter, modified Clark-type polarographic O_2_ microelectrode (OX-10, Unisense A/S, Aarhus, Denmark) and a protective cathode to measure tissue O_2_ levels in different hippocampal layers near the multichannel array. Before the experiment, we calibrated the microelectrode using the manufacturer’s recommended procedure (see Unisense website). Briefly, sensors were immersed into an anoxic solution (zero reading) and then in a solution saturated with O_2_. Since the electrode response to O_2_ is linear, two-point calibration is sufficient. EEG activity was recorded under normoxic (21% O_2_) and hypoxic (16% O_2_) conditions. Field potential and unit activity were recorded with an amplifier and referenced to both internal and cranial references. The data were recorded with a 128-channel TDT system (Tucker-Davis Technologies Inc., Florida, United States) with a sampling frequency of 12 kHz and a LabChart virtual instrument controlling an analogue-to-digital converter card (AD Instruments). The O_2_ sensor was connected to a high-impedance picoammeter (PA 2000, Unisense A/S, Aarhus, Denmark). The signals were A/D converted and recorded in LabChart (AD Instruments). The O_2_ sensing electrode was positioned in proximity (less than 100 μm) to the silicon probes.

#### Microscopy and image editing

Olympus BX61 TRF fluorescent microscope (Olympus Corporation, Tokyo, Japan) was used for light and fluorescent microscopy. Gallyas stains were qualitatively analyzed through light microscopy using a halogen bulb. Fluorescent images were taken using the mercury lamp. All images were taken at x4, x10 or x20 magnifications. Brain slices were immunostained for interneuron markers, imaged using fluorescent microscopy, and mounted using aqueous mounting media, then, coverslips were removed, and brain slices were washed in PBS. Brain slices were re-stained for degenerating ‘dark’ neurons using Gallyas silver staining and imaged using brightfield microscopy. Adobe Photoshop (Adobe Inc., United States) was used to match up hippocampal structures and cells to determine which interneurons were degenerating.

For dark neuron detection, Image-Pro Analyzer v7 (Media Cybernetics) was used. When pixel intensity dropped by at least 50% (typically from 8–9000 to 1–2000) then the structure of interest was considered a silver-stained ‘dark’ neuron.

#### Data processing and statistical analysis

Recordings were processed in Matlab (The MathWorks, Inc., Natick, Massachusetts, United States) using built-in functions to obtain spectral characteristics. Single unit activity was separated based on the online algorithm of the recording software with a bandpass filter of 500–5,000 Hz, yielding firing rate and inter-spike-interval values. Based on the physical location of electrodes, the distance between the recording channels (A4x8-5 mm-100-200-177, NeuroNexus Technologies, Inc., United States), and the amplitude and orientation of the theta waves the position of each recording channel were determined. For analysis, only those unit activities were used which were present both in the 16 and 21% O_2_ levels. Neuron classification (pyramidal or inhibitory cell) was performed based on the physical location of recording channels, firing frequency and inter-spike interval values (Klausberger et al., 2003).

Data were analyzed using Microsoft Excel 365 (Microsoft Inc., Redmond, WA, United States) and SPSS 28 (SPSS Inc., Chicago, IL, United States) for statistical tests and creating graphs. Normality was assessed by the Shapiro–Wilk test. An Analysis of Variance (ANOVA) test was performed if the data were normally distributed. *Post-hoc T*-tests were performed between the individual groups if there was a statistical difference. If the data were non-normally distributed, a Kruskal-Wallis test and Dunn’s multiple comparisons were performed. Bonferroni correction was taken into account where appropriate. We used Student’s paired t-test to compare two variables for the same subject. Data were expressed as mean ± SEM. Confidence values <0.05 were considered to be significant.

## Results

### *In vivo* O_2_ measurement in the hippocampus

We used a 10 μm diameter, modified Clark-type polarographic O_2_ microelectrode to measure tissue O_2_ levels in the hilus-CA1 region. Another probe was in the CA3 region near the multichannel array. When 21% O_2_ was supplied to the mask of the rat (*n* = 10), we measured 20.1 mmHg (SEM = 4.98) tissue O_2_ in the hippocampus, while during 16% O_2_ inhalation, the tissue O_2_ dropped to 8.71 mmHg (SEM = 3.72). Using the Student’s paired *t*-test, we found a significant decrease in the mean tissue O_2_ value (*p* < 0.005) when the O_2_ level was lowered from 21 to 16% in the anaesthetic mask (Figure 1A).

### Assessment of ‘dark’ neurons in the hippocampus

**Figure 2:**
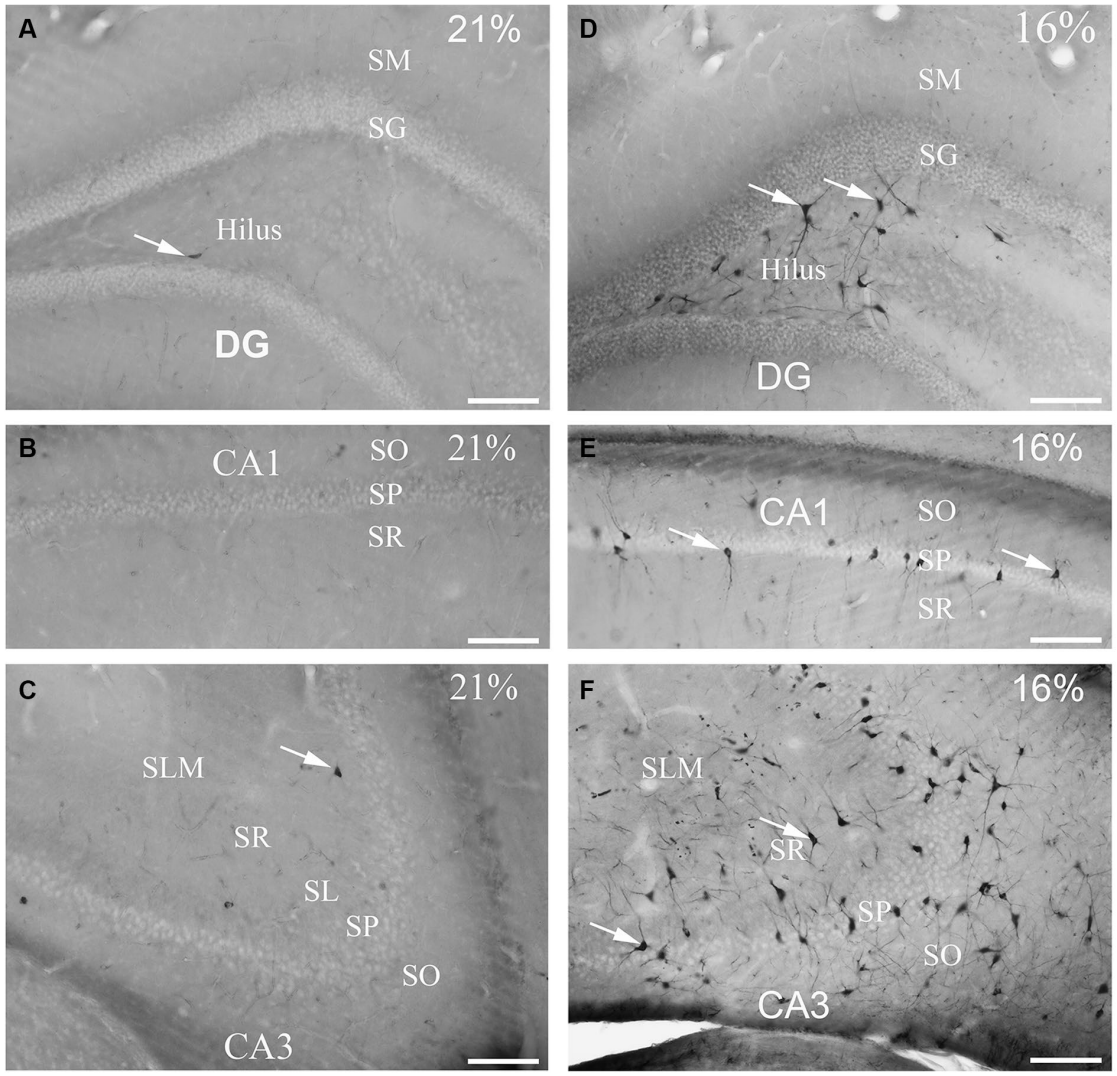
Gallyas silver-stained images of the control (21% O_2_) and hypoxic hippocampi after exposure to 16% O_2_ for 1 h. Very few ‘dark’ neurons are visible in the dentate gyrus **(A)** CA1 **(B)** or the CA3 areas **(C)** in the control hippocampus. **(D)** Numerous neurons are silver-impregnated in the subgranular layer (examples indicated by arrows) and in the hilus in the hypoxic hippocampus. **(E)** Examples of ‘dark’ neurons are pointed by arrows within CA1 str. radiatum exposed to 16% O_2_. **(F)** ‘Dark’ neurons are present in large numbers in the CA3 area with long dendrites descending into the str. radiatum when animals are exposed to 16% O_2_ for 1 h. SO, stratum oriens; SP, stratum pyramidale; SR, stratum radiatum; SL, stratum lucidum; SLM, stratum lacunosum-moleculare; SM, stratum moleculare; SG, stratum granulare; DG, dentate gyrus. Scale bars: 100 μm.

**Figure 3:**
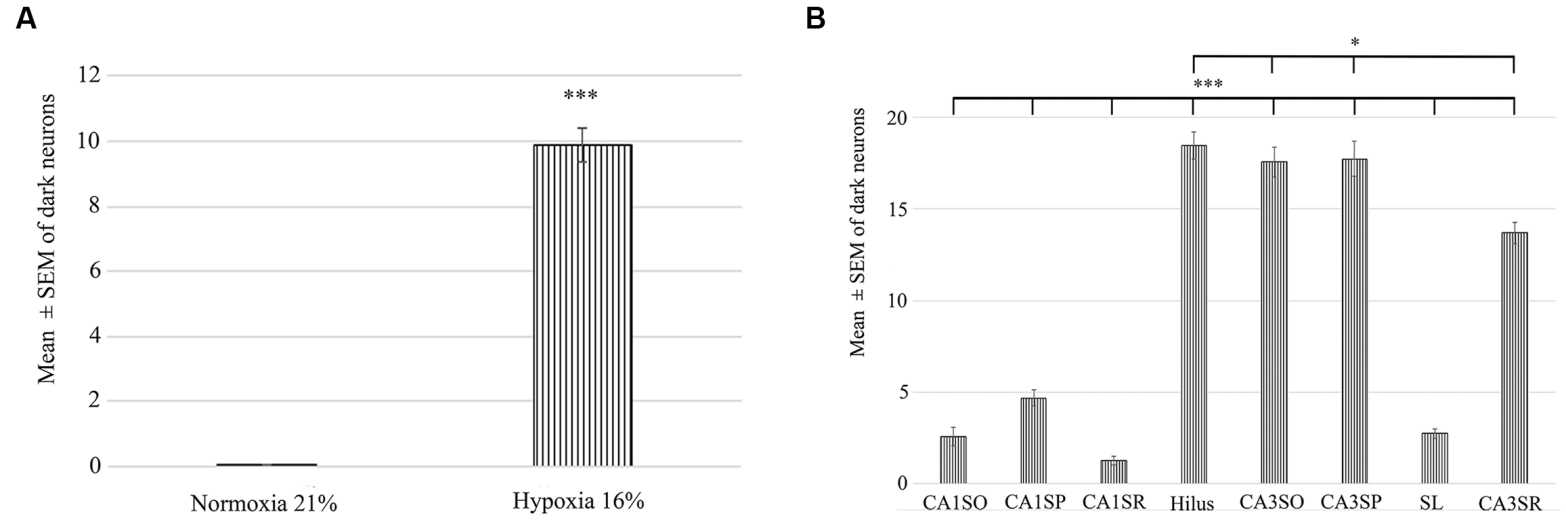
Quantification of the number of dark neurons in the hippocampus in normoxic (21% O_2_) and hypoxic (16% O_2_) conditions. **(A)** There is a statistically significant difference in the number of damaged ‘dark’ neurons between the 21 and 16% experimental groups. **(B)** Area analysis shows that there are fewer ‘dark’ neurons in regions of CA1 than in the hilus and CA3. A statistically significant difference exists between the CA1 str. radiatum, str. pyramidale, str. oriens and CA3 regions, hilus. CA1SO, CA1 stratum oriens; CA1SP, CA1 stratum pyramidale; CA1SR, CA1 stratum radiatum; CA3SO, CA3 stratum oriens; CA3SP, CA3 stratum pyramidale; SL, stratum lucidum; CA3SR, CA3 stratum radiatum. Error bars are represented as mea*n* ± SEM. *p < 0.05 and ***p < 0.001.

To determine the distribution of damaged hippocampal neurons, we analyzed sections processed with the silver impregnation method from each animal. The areas affected by hypoxia are well-classified from previous studies. However, no study has looked at ‘dark’ neurons, which shows damaged neurons at the earliest phase of degradation after short-term mild hypoxia in the hippocampus. One to two ‘dark’ neurons were present in the control animal, which was exposed to 21% O_2_ (Figures 2A–C). In contrast, in hippocampi exposed to hypoxic conditions, there was a moderate quantity of ‘dark’ neurons showing morphological characteristics of inhibitory cells. After 1 h of hypoxia, we found two populations of dark interneurons in the dentate gyrus: one in the subgranular area of the dentate gyrus and the other in the deep hilus (Figure 2D). Numerous ‘dark’ neurons were visible in the CA1 region (Figure 2E) and in the CA3 region, especially in the CA3b (Figure 2F). The quantitative analysis showed a significant difference in the number of ‘dark’ neurons between the normoxic and hypoxic samples (Figure 3A). We also quantified the distribution of ‘dark’ neurons in hypoxic samples in all layers and regions of the hippocampus. In the Cornu Ammonis (CA) we observed silver-impregnated ‘dark’ neurons as follows: most dark neurons were located in CA3 str. Pyramidale (mean = 17.57, SEM = 5.26) and str. Oriens (mean = 17.57, SEM = 4.44), while less was observed in the str. Radiatum (mean = 13.70, SEM = 3.25). In the dentate gyrus, numerous ‘dark’ neurons were found in the dentate hilus (mean = 18.47, SEM = 4.10). In the CA1 layer, most dark neurons were located in the str. Pyramidale (mean = 4.67, SEM = 2.43), and there were fewer in the str. Radiatum (mean = 1.27, SEM = 1.28) and str. Oriens (mean = 2.57, SEM = 2.72). We found fewer, damaged neurons in the CA1 region than in the CA3 region (Figure 3B).

### Immunohistochemical characterization of ‘dark’ neurons

**Figure 4:**
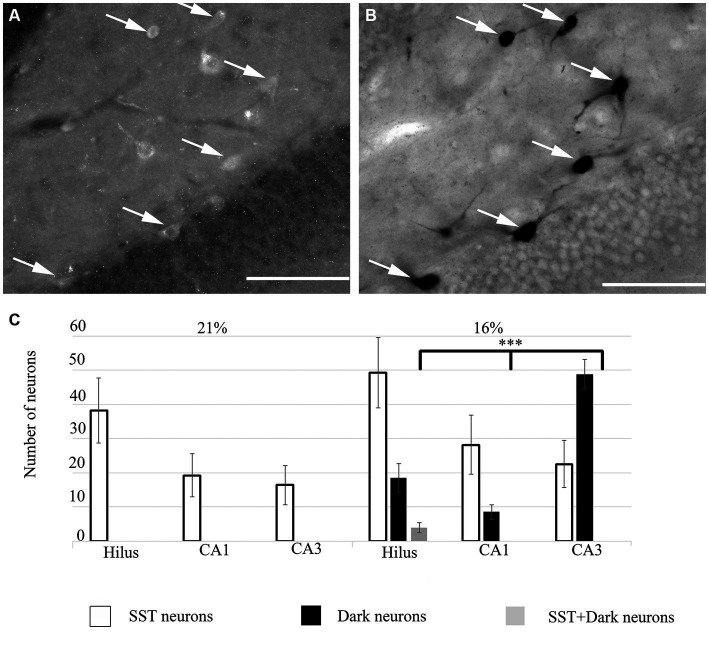
**(A)** Some ‘dark’ neurons in the dentate hilus are SST-immunopositive. Immunofluorescent staining against SST from the 16% O_2_ experimental group shows several immunopositive cells that are also silver-impregnated on **(B)** (arrows). **(C)** Quantitative analysis of SST-immunopositive, ‘dark’ neurons and double-labeled cells in hippocampal regions. Only the hilus contained double-labeled neurons. Scale bars: **(A)** 100 μm, **(B)** 100 μm.

To unveil the neurochemical content of the inhibitory cells, we immunostained the hippocampal sections, photographed the hippocampus and subsequently performed the silver impregnation labeling. We found no double-labeled parvalbumin (PV), neuropeptide Y (NPY), cholecystokinin (CCK), calretinin (CR) or calbindin (CB) immunoreactive inhibitory neurons in the hypoxic sections (not shown). However, somatostatin (SST)-immunoreactive ‘dark’ neurons were present within the hippocampus. A ratio of double-positive cells (dark and SST-immunopositive) to dark cells was quantified. This value was converted to a percentage of ‘dark’ neurons, which were SST-immunopositive. In hypoxic conditions, 23.57% (SEM = 8.8, *n* = 10) of ‘dark’ neurons were SST-immunoreactive cells. The morphology of the double-positive cells varied, indicating heterogeneity of SST-expressing within the hilus (Figure 4) only. Other hippocampal regions (CA1-3) did not contain SST-immunoreactive ‘dark’ neurons.

In summary, when the O_2_ level was lowered from 21 to 16% inhibitory neurons were damaged. Although other subpopulations of inhibitory cells could be among the damaged cells, we were able to identify SST-immunoreactive damaged neurons in the dentate hilus.

### Electrophysiology results

We inserted a 32-channel 4-shank electrode array into the CA1-hilus and CA3b regions of the hippocampus to measure network oscillations and unit activities.

### Network oscillations

**Figure 5:**
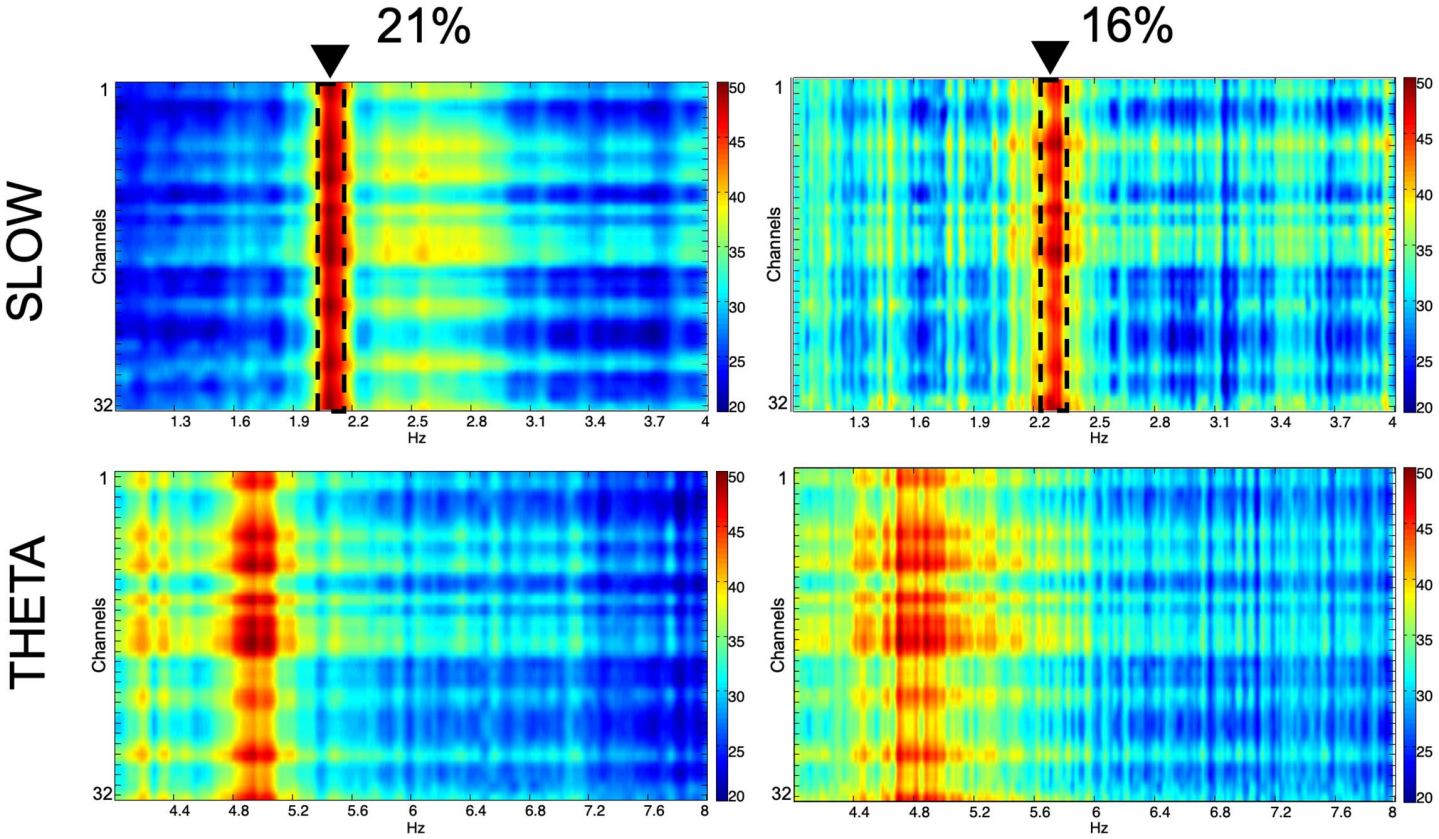
Spectral characteristics of the slow component and the theta oscillation in normoxic (21%) and hypoxic (16%) O_2_ conditions. Colors represent spectral power ranging from blue (low) to red (high) on a common scale (20–50 dB/Hz). The frequency shift of the slow component is demonstrated with dashed lines and black triangles.

**Figure 6:**
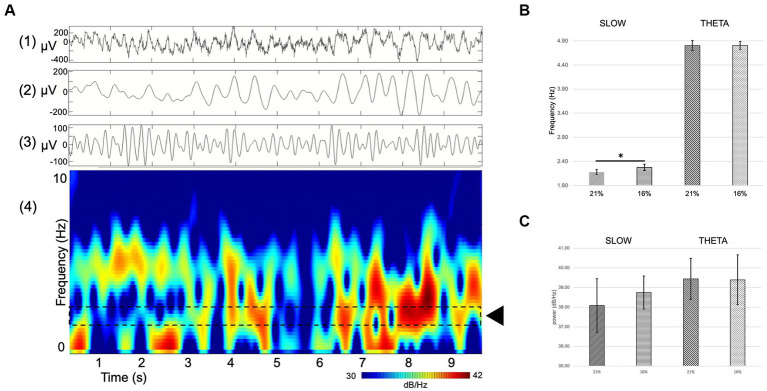
**(A)** Example of single channel hippocampal raw recording (top) and corresponding spectrogram (bottom) displaying theta activity and a slow component. (1) RAW (wideband), (2) slow component filter (1–4 Hz), (3) theta filter (4–8 Hz), (4) spectrogram. Dashed lines delineate the area of interest where the slow component activity can be seen (black rectangle). **(B,C)** Comparison of spectral power (dB/Hz) theta oscillation **(B)** and slow component **(C)** at 21 and 16% O_2_ concentrations. There is a significant shift in the frequency but not in the power of the slow component between normoxic and hypoxic conditions Error bars represent mea*n* ± SEM. *p < 0.05.

We recorded network oscillations in all layers of the hippocampus to determine whether mild hypoxia has a functional effect on neuronal network activity. Most network oscillations that we analyzed (beta, gamma) showed no significant change in hypoxic conditions (not shown). However, the peak frequency of a recurring low-frequency activity, peaking at around 2.18 Hz, shifted significantly when the O_2_ concentration was changed. The peak frequency at 21% O_2_ level is 2.18 Hz (SEM = 0.05), while at 16% O_2_ level, it shifts to 2.28 Hz (SEM = 0.07). Student’s paired t-test demonstrated that the change in peak frequency is significant (*n* = 9, confidence level: 0.05). When we compared this slow activity to the theta oscillation, we found that there is no significant change in the theta frequency when the O_2_ concentration is modified (at 21% 4.8 Hz, SEM = 0.11, while at 16% 4.81 Hz, SEM = 0.08, *n* = 9), thus the peak frequency change is a unique feature of the slower frequency activity (Figures 5, 6).

The spectral power of the slow component as well as the theta oscillation was computed to compare not only changes in frequency but also in power. Slow component spectral power values show no statistically significant changes (21% O_2_: 38.08 ± 1.37 dB/Hz vs. 16%: 38.74 ± 0.84 dB/Hz, mean and SEM). Theta oscillation spectral power shows no significant change either (21% O_2_: 39.44 ± 1.05 dB/Hz vs. 16%: 39.4 ± 1.27 dB/Hz, mean and SEM) (Figures 6B,C).

To summarize the network oscillation results, we found that the reduction of O_2_ concentration induces a selective and significant shift in the frequency of a slow field potential activity while keeping other oscillations unchanged and maintaining the same power in the spectrum.

### Unit activity

**Figure 7:**
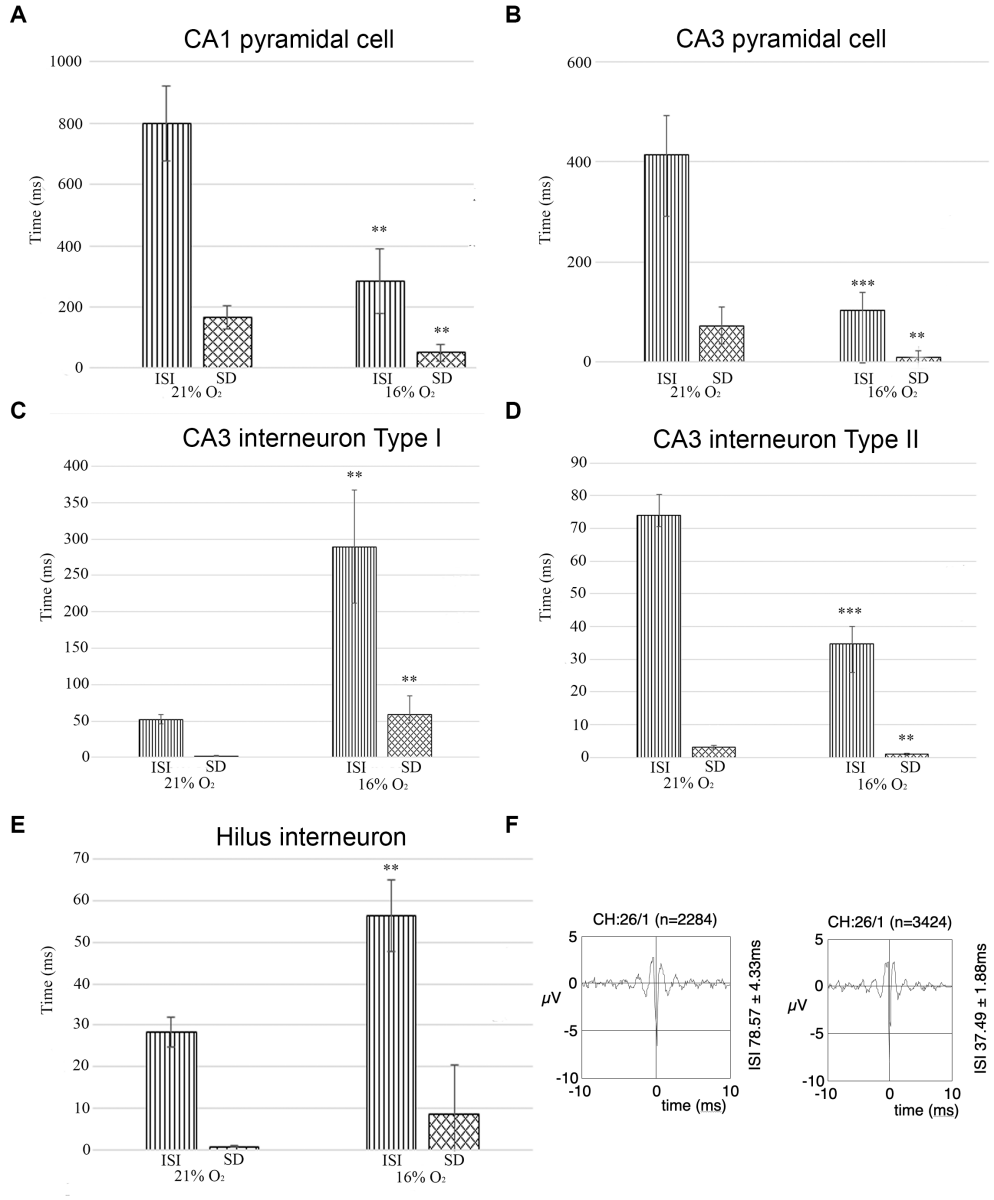
**(A)** The unit activity of pyramidal cells in the CA1 region in hypoxic and normoxic conditions. Inter-spike interval (ISI) and standard deviation values significantly decreased in a hypoxic environment meaning that hypoxia increases firing frequency and CA1 pyramidal cells fire more regularly. **(B)** Pyramidal cells in the CA3 region change activity in hypoxia compared to normoxic conditions. Inter-spike interval (ISI) and standard deviation values were analyzed. Based on the analysis, the frequency of the action potential of the hypoxic CA3 pyramidal cell increased (ISI decreased) and pyramidal cells fired more regularly (SD decreased). **(C)** The activity of one group of putative inhibitor interneurons in the CA3 region in hypoxic and normoxic environments. Inter-spike interval (ISI) and standard deviation values were analyzed Based on the analysis, the frequency of the action potential of hypoxic interneurons decreased (ISI increased) and fired more irregularly (SD increased). **(D)** The activity of another group of putative inhibitory interneurons in the CA3 region. The frequency of the action potential of hypoxic interneurons increased (ISI decreased) in a hypoxic environment and the activity showed more regularity (SD decreased). **(E)** Unit activity of hilar interneurons in normoxic and hyperoxic conditions has been compared based on ISI and SD values. ISI values significantly increased indicating that the activity of hypoxic interneurons decreased. **(F)** Example epoch of a single unit comparing its firing characteristics due to change in O_2_ concentration (21% on the left, 16% on the right). The firing rate (ISI 78.57 ms at 21% O_2_ and 37.49 ms at 16% O_2_) increased but not the shape of the unit. Error bars represent mea*n* ± SEM. **A**, **p < 0.005; **B**, **p < 0.005 and ***p < 0.001; **C**, **p < 0.005; **D**, **p < 0.005 and ***p < 0.001; **E**, **p < 0.005.

Interneuron and pyramidal cell unit activity have been separated based on the inter-spike interval (ISI) and standard deviation values for further statistical analyses [see Materials and methods (Klausberger et al., 2003)]. We recorded unit activity in normoxic conditions and when we achieved stable unit recording, we lowered the inhaled O_2_ to 16% and continued recording the same neurons. In parallel, we measured the brain tissue O_2_ level near the recording electrodes (see above) to make sure that the change in local O_2_ level caused the change in firing frequency. Seven stable pyramidal cells were found in the CA1 with a mean ISI value of 800.15 ms (SEM = 122.29) and mean SD of 166.69 ms (SEM = 36.99) at 21% O_2_ concentration, compared to the ISI of 284.67 ms (SEM = 106.26) and SD of 50.17 ms (SEM = 28.73) while 16% O_2_ was supplied. When the O_2_ level was lowered from 21 to 16%, we detected a significant decrease in the mean ISI value (confidence level: 0.006) as well as in the SD value (confidence level: 0.008) (Figure 7A; Table 1). Twenty-two stable pyramidal cells (*n* = 22) were found in the CA3 with a mean ISI value of 418.2 ms (SEM = 78.6) and mean SD of 72.74 ms (SEM = 28.11) at 21% O_2_ concentration, compared to the ISI of 103.92 ms (SEM = 34.58) and SD of 8.71 ms (SEM = 4.78) while 16% O_2_ was supplied. When the O_2_ level was lowered from 21 to 16%, we detected a significant decrease in the mean ISI value (confidence level: 0.0000) as well as the SD value (confidence level: 0.004) (Figure 7B; Table 1). Thirty-five interneurons with stable recording in both 21 and 16% O_2_ conditions were identified in the CA3 region, which was divided into two groups. The first interneuron group (*n* = 19) had a mean ISI value of 52.34 ms (SEM = 6.58) and mean SD of 2.06 ms (SEM = 0.46) when 21% O_2_ was supplied, compared to the ISI of 289.12 ms (SEM = 77.71) and SD of 58.78 ms (SEM = 26.0) during 16% O_2_ concentration. Using Student’s paired t-test, we found a statistically significant increase in the mean ISI value (confidence level: 0.007) as well as the SD value (confidence level: 0.002) when the O_2_ level was lowered from 21 to 16% (Figure 7C; Table 1). In contrast to the first interneuron group, the second group (*n* = 16) ISI value (74.00 ms, SEM = 6.29) and mean SD (3.14 ms, SEM = 0.47) significantly decreased to ISI 34.65 ms (SEM = 5.37) and SD of 1.00 ms (SEM = 0.25) when O_2_ level was lowered from 21 to 16% (Figure 7D; Table 1). Ten interneurons were identified in the hilus region with a mean ISI value of 28.24 ms (SEM = 3.53) and mean SD of 0.75 ms (SEM = 0.21) when 21% O_2_ was supplied, compared to the ISI of 56.31 ms (SEM = 8.61) and SD of 13.94 ms (SEM = 11.8) during 16% O_2_ concentration. Using Student’s paired *t*-test, we found a statistically significant increase in the mean ISI value (confidence level: 0.003) when the O_2_ level was lowered from 21 to 16% (Figure 7E; Table 1). When the characteristics of a single unit were analyzed over time both in 21 and 16% O_2_ environment, we found no change in shape or amplitude of the analyzed and tracked single units (Figure 7F).

**Table 1:** Summary of neuronal firing rates in normoxic and hypoxic conditions.

Neuron type	21% O_2_ ISI (mean + SD) ms	16% O_2_ ISI (mean + SD) ms	Frequency change in hypoxia	Firing regularity change in hypoxia
CA1 pyramidal	800.15* ±166.69*	284.67* ±50.17*	Increase	Regular
CA1 interneuron	nd	nd	nd	nd
CA3 pyramidal	418.2* ± 72.74*	103.92* ± 8.71*	Increase	Regular
CA3 interneuron Type I	52.34* ±2.06	289.12* ±58.78	Decrease	Irregular
CA3 interneuron Type II	74.00* ± 3.14*	34.65* ±1.00*	Increase	Regular
Hilus	28.24* ±0.75*	56.31* ±13.94*	Decrease	Irregular

Asterisks represent significant differences between the two conditions. A decrease of SD means more regular activity of neurons. (nd, not detected).

We conclude that while the activity of pyramidal cells increased both in the CA1 and CA3 regions, the change of activity of inhibitory cells was more heterogeneous. The change in SD of ISI indicates a more regular firing in the case of CA1 and CA3 pyramidal cells, while CA3 Type I and hilar interneuron activity became more irregular in 16% inhaled O_2_ concentration while Type II CA3 interneurons fired more regularly in hypoxic conditions.

## Discussion

In this study, we investigated the effect of acute mild hypoxia on the hippocampal network using anatomical and physiological methods. We detected numerous compacted silver-labeled inhibitory neurons in all hippocampal regions of the rat. Many inhibitory neuron subgroups are present in the hippocampus with different functions to shape network oscillation and participate in the formation of memory traces (i.e.: Sik et al., 1994, 1995, 1997; Freund and Buzsáki, 1996). The main role of interneurons is to control and synchronize the activity of excitatory pathways. When the activity of inhibitory cells decreases either due to cell death, change in excitatory input onto the inhibitory cells, or intrinsic properties (i.e.; channel or membrane properties) the balance of excitatory-inhibitory activity turns that can result in pathological conditions such as epilepsy and seizures (Kepecs and Fishell, 2014).

The presence of ‘dark’ neurons has been described in animal models of several neurological diseases, such as hypo- and hyperglycemia, and status epilepticus (Agardh et al., 1981; Atillo et al., 1983; Söderfeldt et al., 1983; Auer et al., 1985; Gallyas et al., 2008). In the case of reperfusion after focal ischemia, this change can be observed in the marginal areas of the ischemic focus (Kalimo et al., 1982; Kirino, 1982; Nedergaard and Diemer, 1988; Sillesen et al., 1988; Czurkó and Nishino, 1993; Hsu et al., 1994). In four-vessel occlusion ischemia, ‘dark’ neurons are formed in the CA1 region and the hilus of the hippocampus (Kirino, 1982; Czurkó and Nishino, 1993; Hsu et al., 1994).

Ultrastructural investigation using an electron microscope showed that freshly formed ‘dark’ nerve cells appear intact but have ultrastructural compaction (Gallyas et al., 2004). The cell volume of the affected cells is reduced by about half without the plasma membrane rupturing, due to the physicochemical gel–gel transformation immediately spread throughout the intraneuronal space, resulting in a perturbed structure characterized by hyperargyrophilia, hyperbasophilia and high electron density. The cisternae of the endoplasmic reticulum (ER) contract, while the Golgi cisternae expand, while the volume of mitochondria and multivesicular bodies does not change (Gallyas et al., 2004, 2006). A portion of the affected neurons regains their original morphology and likely recovers within a few hours, while another portion dies (Gallyas et al., 2005; Toth et al., 2016). The compacted state of the neurons likely means functional impairment, which may persist for some time even after the regeneration of the cells.

Subclasses of inhibitory neurons can be visualized using inhibitory neuron markers, such as NPY, SST, CCK, PV, CB, CR, NOS, VIP etc. Although some of the markers are expressed in the same neurons (i.e.: CR-SST, SST-NOS, NPY-SST), many (i.e.: PV, CB, CR) can be used as distinctive markers for visualizing inhibitory neuron subgroups (Greenwood et al., 1981; Baimbridge and Miller, 1982; Kohler and Chanpalay, 1982; Morrison et al., 1982; Chronwall et al., 1985; Kohler et al., 1986, 1987; Kosaka et al., 1987; Jacobowitz and Winsky, 1991; Valtschanoff et al., 1993; Freund and Buzsáki, 1996). We found that only a small portion of SST-immunoreactive inhibitory cells in the dentate hilus is vulnerable to short-term mild hypoxia. In the hilus, a subpopulation of interneurons is vulnerable to overexcitation causing Ca^2+^-induced oedemia and cell death (Hsu and Buzsaki, 1993; Maglóczky and Freund, 1995), thus it is likely, that a similar mechanism is the cause of the SST-expressing interneuron damage in the hilus in mild acute hypoxia. SST-immunopositive neurons in the hippocampus are all GABAergic (Somogyi et al., 1984; Kosaka et al., 1988), with 14% of all inhibitory interneurons being SST positive (Kosaka et al., 1988). In the dentate gyrus, SST-immunopositive neurons are located predominantly in the hilus, the majority in the subgranular zone (Freund and Buzsáki, 1996). In the subgranular zone, SST cells have a fusiform soma and their dendrites run parallel to the str. Granulosum. These are predominantly described as hilar interneurons with perforant pathway-associated axon terminals (HIPP) interneurons (Freund and Buzsáki, 1996). HIPP cells target PV-containing perisomatic basket cells, where they control the basket cell activity (Savanthrapadian et al., 2014). Thus, the decrease of the SST-immunoreactive neurons can lead to a decrease in the activity of excitatory granule cells of the dentate gyrus. Since we were unable to record unit activity from granule cells, we can only hypothesize the physiological consequences.

It also needs to be mentioned that the lack of immunoreactivity of ‘dark’ neurons does not necessarily mean the lack of protein expression that distinguishes inhibitory neuron subgroups. Because of the harsh treatment necessary to perform the silver impregnation, organic materials can easily deteriorate. For this reason, we performed the immunoreaction first, and then we developed the section for ‘dark’ neuron labeling. Although this protocol ensured that the chemical treatment did not destroy antigens, it is feasible that deterioration of cell structure including protein degradation caused by hypoxia cause underestimation of immunoreactive inhibitory cells. The immunoreactivity of PV and CB inhibitory cells remains after ischemia and only disappears from the somata after 4th postischemic day (Johansen et al., 1990). Although after 1 h of the hypoxic event, the ‘dark’ stained neurons were predominantly present in the str. Pyramidale with dendrites descending into the str. Radiatum suggesting that these may be PV-immunopositive basket cells, we still conclude that short-term mild hypoxia does not likely cause structural changes in these two populations of inhibitory cells based on their demonstrated resistivity to hypoxia (Johansen et al., 1990).

The sensitivity of the hippocampus to hypoxia does not necessarily show the same distribution of damaged or dead neurons as in ischemia. For example, normobaric hypoxia causes significant morphological changes in cells of the CA3 region, while granule cells in the dentate gyrus are less severely affected, whereas neurons in the CA1 region are mostly resistant to up to half an hour of hypoxic damage (Yamaoka et al., 1993). Similarly, hypobaric hypoxia can severely damage hippocampal neurons, causing morphological changes, neurodegeneration, and apoptosis, to a greater extent in the CA3 than in the CA1 area (Maiti et al., 2007).

The firing rates of pyramidal versus inhibitory cells are considerably different especially under anaesthesia when place cells are not bursting [for review see (Klausberger et al., 2003)]. We used both the position of recording channels and the firing characteristics of the neurons to distinguish excitatory cells from inhibitory neurons. Under anaesthesia, pyramidal cells fire at a low frequency and without burst activity, while inhibitory neurons fire at a high frequency and often in a bursting fashion. We used 200 ms for the ISI as a cut-off point to distinguish pyramidal cells from inhibitory neurons in the pyramidal cell layers.

Hypoxic conditions induce a decrease in adenosine triphosphate (ATP), a rise in cytoplasmic free calcium, and an accumulation of extracellular adenosine (produced by ATP breakdown). This causes a disturbance in ion balance, which leads to the early cessation of electrical activity (‘firing’) and the disappearance of excitatory synaptic potentials (Krnjević, 1999). It is well-known, that the majority of neurons are sensitive to hypoxia, however, the different neuron types can react differently even within the same brain region (Kawasaki et al., 1990; Haddad and Jiang, 1993; Peña and Ramirez, 2005). For hippocampal neurons, hypoxia can cause either hyperpolarization or depolarization (or an initial depolarization followed by hyperpolarization or vice versa), leading to the inactivation of transient ion channels (Fujiwara et al., 1987; Leblond and Krnjevic, 1989; Haddad and Jiang, 1993; Fujimura et al., 1997; Englund et al., 2001). It was reported that neuronal excitability decreased in the CA1 region during hypoxia-inflammation, which is probably explained by the strong expression of K_ATP_ channels in CA1 neurons and excitability are at least partially regulated by the availability and voltage dependence of K_V_ channels (Zawar et al., 1999; Sun and Feng, 2013; Yang et al., 2018, 2021). In our hypoxia model, we detected a similar decrease in electrical activation of hilus interneurons during a brief, normobaric hypoxic period. SST-immunoreactive interneurons in the hilus are known to be particularly sensitive to ischemia and hypoxia induces a presynaptic inhibition of excitatory input to dentate interneurons mediated in part by activation of metabotropic glutamate receptors (Johansen et al., 1987; Matsuyama et al., 1993; Doherty and Dingledine, 1997). Hypoxia-induced hyperpolarization in hippocampal pyramidal cells is often mediated by Ca^2+^-dependent K^+^ channels (Leblond and Krnjevic, 1989; Erdemli et al., 1998; Nowicky and Duchen, 1998). We found that the firing frequency of pyramidal cells increased in response to short-term hypoxia in the CA1 and CA3 regions. This suggests that hippocampal pyramidal cells are depolarized by hypoxia. Hypoxia has been observed to inhibit several potassium channels (voltage-gated and TWIK-related acid-sensitive K^+^ (TASK)), leading to membrane depolarization and the influx of Ca^2+^- through L-type channels (Buckler et al., 2000; Plant et al., 2002; Campanucci et al., 2003; Weir and Olschewski, 2006). Interneurons were divided into two groups based on their firing frequency (type I and type II). We observed that the electrical excitability of type I interneurons in the CA3 region dropped in hypoxia. Based on previous studies, inhibitory synapses are particularly sensitive to hypoxia, and hypoxic hyperpolarization is often significant in the population of inhibitory interneurons (Khazipov et al., 1993; Doherty and Dingledine, 1997). In our study, we observed an increase in the activity of another group of CA3 interneurons (type II) confirming the functional heterogeneity of hippocampal interneurons.

Previous studies have shown that the delta band power of hippocampal network oscillation increases during ischemic hypoxia in both rats and human subjects (Fanciullacci et al., 2017; Ferreira et al., 2021). In the present study, we observed a pronounced low-frequency activity (2.18 Hz) in the delta wave that increased with normobaric hypoxia under urethane anaesthesia in the hippocampus. On the other hand, we found no changes in the frequency of the theta, beta or gamma bands. Delta wave activity can arise in the thalamic neurons and the deep cortical layers (Dossi et al., 1992; Steriade et al., 1993). It is known that blood flow has a direct relationship with delta wave activity. If the decrease in blood flow exceeds the ischemic threshold of 18 mL/100 g/min, the delta wave activity gradually increases (Foreman and Claassen, 2012). Pyramidal neurons found in the cortical III, V, and VI layers are especially sensitive to decreased blood flow (Jordan, 2004). Based on this observation, an increase in delta activity may represent the sustained hyperpolarization and inhibition of the cortical neurons, which influence the activity of the hippocampus via the entorhinal cortex (Sirota et al., 2003; John and Prichep, 2006; Fanciullacci et al., 2017). In our case, the decrease in blood flow is unlikely, as it is known that the reduced oxygen supply to the brain results in several compensatory mechanisms, for example, increased cerebral blood flow (Kety and Schmidt, 1948; Kuwahira et al., 1993; Xu et al., 2012; Ogoh et al., 2014). However, it is important to mention that mild hypoxia impairs autoregulation, thus affecting the regulation of the blood flow (Iwasaki et al., 2007; Nishimura et al., 2010; Katsukawa et al., 2012). Furthermore, urethane anesthesia may have altered the neurotransmission (Shirasaka and Wasterlain, 1995; Sceniak and Maciver, 2006), thereby making neurons more sensitive to the response to hypoxia. It also needs to be noted that the frequency and power of network oscillations of the hippocampus are modified by anaesthetics. The frequency of oscillations (slow wave, theta oscillation) is significantly higher in behaving than animals under anaesthesia (Vandecasteele et al., 2014) and for example gamma power decreases in isoflurane anaesthesia (Hudetz et al., 2011). We predict that in freely behaving rodents the observed slow oscillation frequency is higher, but the frequency shift caused by mild acute hypoxia remains prominent. Whether the power of the slow oscillation remains unchanged requires additional experiments that is outside of the focus of our current study.

In conclusion, the results of the present study suggest that mild normobaric hypoxia has a significant effect on the viability of hippocampal inhibitory neurons, mainly SST-immunopositive neurons. Mild hypoxia increases the firing activity of CA1 and CA3 pyramidal neurons and causes changes in delta oscillation. Neuronal loss or dysfunction can affect the balance between excitatory and inhibitory neurons, affecting network activity, thereby impairing learning abilities and reducing plasticity. Possible neuronal damage and altered information processing caused by short-term mild hypoxia can lead to neurochemical and neurophysiological disorders.

## Data availability statement

The raw data supporting the conclusions of this article will be made available by the authors, without undue reservation.

## Ethics statement

The animal study was approved by the National Ethical Council for Animal Research, Hungary. The study was conducted in accordance with the local legislation and institutional requirements.

## Author contributions

AH: Data curation, Formal analysis, Investigation, Methodology, Writing – original draft. AM: Formal analysis, Investigation, Methodology, Software, Visualization, Writing – original draft. CT: Data curation, Formal analysis, Visualization, Investigation, Writing – original draft. KK: Data curation, Formal analysis, Investigation, Methodology, Writing – original draft. GS: Methodology, Writing – review & editing. JP: Data curation, Investigation, Methodology, Supervision, Writing – original draft. AS: Conceptualization, Funding acquisition, Project administration, Resources, Supervision, Writing – review & editing.
